# Parasitism perturbs the mucosal microbiome of Atlantic Salmon

**DOI:** 10.1038/srep43465

**Published:** 2017-03-07

**Authors:** M. S. Llewellyn, S. Leadbeater, C. Garcia, F.-E. Sylvain, M. Custodio, K. P. Ang, F. Powell, G. R. Carvalho, S. Creer, J. Elliot, N. Derome

**Affiliations:** 1 School of Life Sciences, University of Glasgow, Glagsow, UK; 2 St Andrew’s Marine Station, Department of Fisheries and Oceans, New Brunswick, Canada; 3 Universite Laval, Quebec, Canada; 4 Universidade Federale do Rondonia, Porto Vehlo, Brazil; 5 Cooke Aquaculture, Canada; 6 Marine and Fisheries Genetics Laboratory, University of Wales, Bangor, Wales, UK

## Abstract

Interactions between parasite, host and host-associated microbiota are increasingly understood as important determinants of disease progression and morbidity. Salmon lice, including the parasitic copepod *Lepeophtheirus salmonis* and related species, are perhaps the most important problem facing Atlantic Salmon aquaculture after feed sustainability. Salmon lice parasitize the surface of the fish, feeding off mucus, scales and underlying tissue. Secondary bacterial infections are a major source of associated morbidity. In this study we tracked the diversity and composition of *Salmo salar* skin surface microbiota throughout a complete *L. salmonis* infection cycle among 800 post-smolts as compared to healthy controls. Among infected fish we observed a significant reduction in microbial richness (Chao1, P = 0.0136), raised diversity (Shannon, P < 7.86e-06) as well as highly significant destabilisation of microbial community composition (Pairwise Unifrac, beta-diversity, P < 1.86e-05; P = 0.0132) by comparison to controls. While undetectable on an individual level, network analysis of microbial taxa on infected fish revealed the association of multiple pathogenic genera (*Vibrio, Flavobacterium, Tenacibaculum, Pseudomonas*) with high louse burdens. We discuss our findings in the context of ecological theory and colonisation resistance, in addition to the role microbiota in driving primary and secondary pathology in the host.

New data from epidermal (e.g. ref. 1) and intestinal (e.g. refs 2, 3, 4) parasitic disease systems suggest major roles for host-associated microbiota in promoting effective host immunity (e.g. ref. 1) or driving host pathology^2^. More widely, commensal microbiota – especially in the gut - are known to exert ‘colonization resistance’ on potential opportunistic pathogens, inhibiting over-growth and pathogenesis (e.g. ref. 5). In aquatic systems major pathogens and putative opportunists are frequently identified as asymptomatic infections. As such, the host-associated microbiome can act as the source of many disease agents which emerge as important pathogens under the right conditions^6^.

Salmon lice are copepod ectoparasites of salmon. Several species of the two main genera, *Lepeophtheirus* and *Caligus*, are distributed globally and infect both Pacific and Atlantic salmonid species^7^. Costs and losses attributed to sea louse infection, estimated at €300M million annually, are the single greatest pathogen burden on the global salmonid aquaculture industry^8^. In the North Atlantic, a single species predominates (*Lepeophtheirus salmonis*), causing year-round infestations of Atlantic Salmon (*Salmo salar*) housed in marine cages, with concomitant ramifications for fish health as well as aquaculture economics and sustainability.

*L. salmonis* are the cause of substantial morbidity in their salmonid hosts. Pathology arises primarily through louse feeding behaviour whereby their rasping maxillae scrape mucus, scales, and underlying tissue into their digestive tract^9^. Osmoregulatory dysfunction, fluid and blood loss result. There is evidence that *L. salmonis* secrete several proteases to assist with feeding^10^. A further significant source of host pathology is secondary bacterial infections (e.g. *Aeromonas salmonicida* and *Piscirickettsia salmonis*, among others) that often accompany salmon lice infection^9^,^11^,^12^. In addition to breaking down physical barriers, it is thought that *L. salmonis* secrete immunosuppressive chemicals (e.g. Prostaglandin E, Trypsin), that down-regulate T-cell and other functions required to effectively cope with bacterial pathogens^13^,^14^. Experimental *S. salar* co-infections between a Chilean copepod species *Caligus rogercresseyi* and the bacteria *P. salmonis* show that survival rates in the co-infected fish (0% after 53 days) are substantially lower than in fish infected with *P. salmonis* alone (c.57% over the same period)^11^. Whilst there is an increasing understanding of salmonid immunity to copepod pathogens, as well as to secondary agents, nothing is known about the role commensal microbes may play in such infections.

In this study we set out to explore the evolution of the host epidermal microbial community during *L. salmonis* infection of marine-phase *S. salar* raised in aquaculture conditions. We aimed to assess the extent of association between features of the epidermal microbiome and different intensities of parasite burden. To achieve this we employed 16S rRNA amplicon deep sequencing on the epidermal mucosa of a subset of 1200 *S. salar* post smolts (800 tests, 400 controls) experimentally infected with *L. salmonis*. Substantial perturbation of microbial community structure and composition was observed in infected fish, consistent with a reduction in the ‘colonization resistance’ of the system. Network analysis suggested a correlation with increasing louse load and multiple potential pathogens. Together, our data highlight the role of parasite-perturbed host associated microbiota as drivers of disease, as well as new potential targets for therapeutic interventions.

## Results

### Experimental infection outcomes

**Figure 1:**
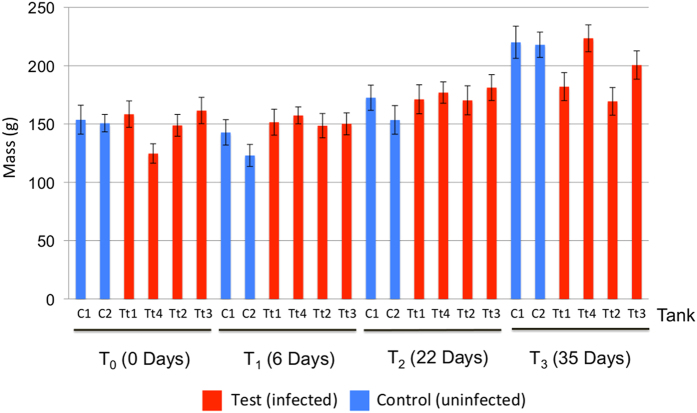
Impact of *Lepeophtheirus salmonis* infection on salmon growth during the experiment. Mean values for fish mass with error bars showing +/− standard error are shown per tank and time point in test and control tanks. An analysis of variance indicates a borderline insignificant impact of infection on fish mass across all six tanks (P = 0.082), and significant when only the four tanks (Test (Tt)1, Test(Tt) 2,C1,C2) from which longitudinal microbiome sampling had occurred (P = 0.0007).

Exposure of post-smolts to 40 *L. salmonis* copepodids/fish resulted in final louse counts ranging between two and 67 adult lice per individual (See frequency distribution in Figure S1). Significant differences in louse load (ANOVA, P = 0.0035) were noted between tanks (Figure S2). Weight gain differences were noted between some infected and control tanks at T_3_ (Fig. 1). A mixed-model incorporating tank as a random effect showed a clear effect of lice on fish weight overall (Fig. 1, P = 0.00679). Only mucus samples from Test tank 3 & Test tank 4 were only 16S rRNA sequenced at the final time point (T_3_), a decision taken prior to and weight/growth calculations. For the four test tanks, where individual fish were recaptured on multiple samplings, individual growth rates (mass change (g) day^−1^) were calculated (mean: 1.118 g day^−1^, range: −1.57 to 3.55). No correlation was observed between individual growth rate and louse load (Linear regression, P > 0.05, R^2^ = 0.01667). Among the 50 salmon families included in our study (all survivors), no impact of family was noted on louse density (ANOVA, P = 0.425). For the infected fish for which we could determine individual growth rate (N = 36), no effect of family on growth rate was detected.

### Microbial alpha and beta diversity destabilisation in response to *L. salmonis* infection

**Figure 2:**
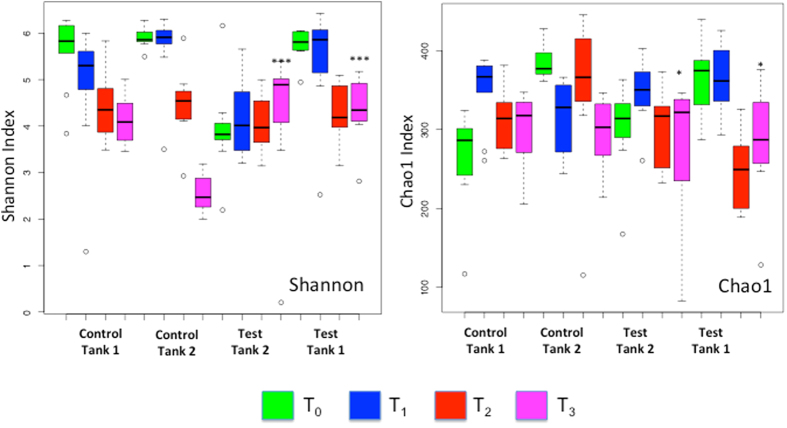
Alpha diversity (Shannon) and richness (Chao1) variation in *Salmo salar* skin mucosal microbiota in response to infection with the sea louse *Lepeophtheirus salmonis*. Box plots show diversity and richness profiles at each sampling point T_0_–T_3_. A significant decline in Chao1 richness (Fig. 2) was noted between test and control tanks at T_2_ (P = 0.0136). Shannon diversity increased at T_3_ (P < 7.86e-06). *Denotes significance level.

**Figure 3:**
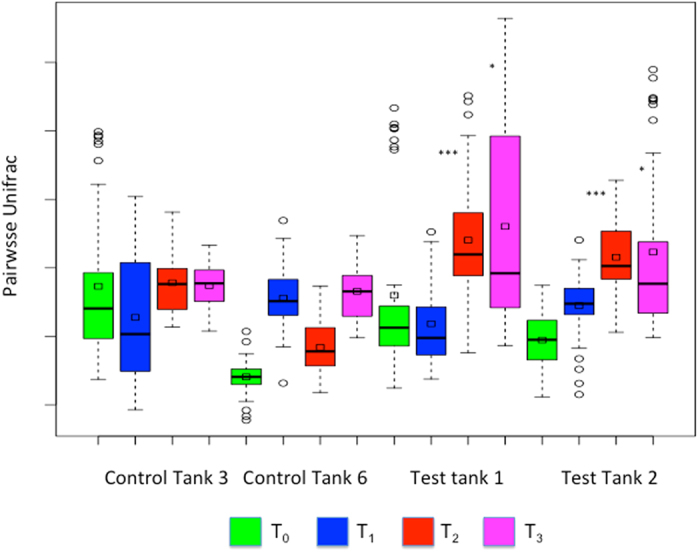
Pair-wise beta diversity measurements show destabilisation of *Salmo salar* skin mucus bacterial assemblages in response to infection with the sea louse *Lepeophtheirus salmonis*. Box plots indicate variation in inter-sample pairwise Unifrac distance per tank and sampling point T_1_–T_4_. Significant increases in inter-sample variation was noted at the Times 2&3 between control and infected tanks (T_2_ P < 1.86e-05 T_3_, P = 0.0132).

**Figure 4:**
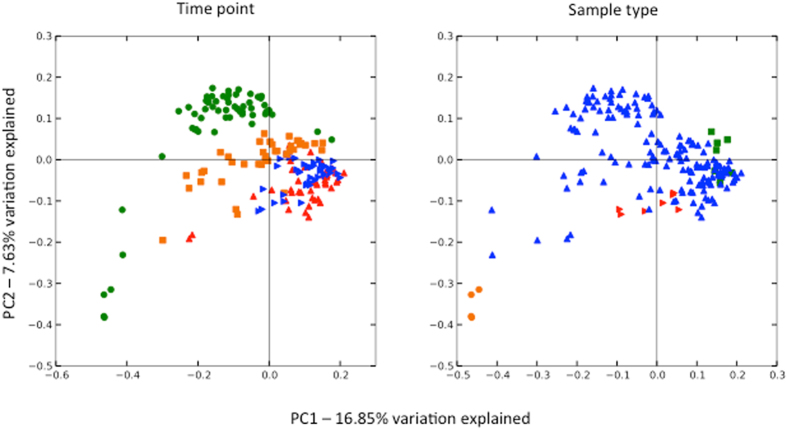
Relationship between microbiota sampled from *Salmo salar, Lepeophtheirus salmonis* and environmental samples (water, biofilm) over the course of experimental infection. A composite multidemensional scaling (MDS) plot of sample clustering is based on a single principal coordinates analysis (PCoA) of pairwise un-weighted Unifrac distances between all samples. The left hand plot figure depicts samples coloured by time point (T_0_ = red, T_1_ = blue, T_2_ = orange, T_3_ = green). The right hand plot depicts the same plot coloured by sample type (skin mucous = blue, water samples = green, tank biofilm = red, lice = orange).

After error filtering, alignment and chimera removal, a total dataset of 4,512,783 reads was generated across all samples which clustered into 1754 97% OTUs (for sample numbers, see Supplementary Information). This dataset was then rarefied to 13,700 reads per sample and low abundance OTUs filtered out (<100 total). Rarefaction curves confirmed saturation at this depth across the dataset (Figure S3). Again treating tank as a random effect, alpha diversity (Shannon) and richness (Chao1) were compared across test and control tanks and sampling points. A significant decline in Chao1 richness (Fig. 2, P = 0.0136) was noted between test and control tanks at T_2_ but a significant increase in Shannon diversity at T_3_ (Fig. 2, P < 7.86e-06). (Models: Chao1~Time * Treatment + (1 | Tank); Shannon~Time * Treatment + (1 | Tank)). Very strikingly, we noted strong evidence for beta-diversity destabilisation of host mucosal microbiota in fish infected by pre-adult (T_2_) and adult lice (T_3_) (Fig. 3, T_2_ P < 1.86e-05 T_3_, P = 0.0132; Model: Beta_Div~Time * Treatment + (1 | Tank)). No significance was obtained for treatment (infected or not) at earlier time points. Destabilisation can be clearly observed in the principal coordinates analysis based on Unifrac distances displayed in Fig. 4. As is observable from Fig. 3, destabilisation involves an increase in the mean beta-diversity and its variance with time. As such, beta-diversity in both test tanks experienced a ‘shot-gun’ spread of increasing dissimilarity over the course of infection, compared to the two control tanks. As well as the important role of time in defining microbiome composition, other features of interest in Fig. 4 include clear clustering of all water samples (T_0–3_) with all mucus samples at T_0&1_ (Fig. 4, Panel B). By contrast, biofilm samples were distributed more widely across different time points (Fig. 4, Panel E). Samples taken from pooled *L. salmonis* intestines were highly divergent with respect to their microbial composition (Pairwise Unifrac, Fig. 4, Panel F), although fairly similar among tanks. Multivariate permutational analysis of beta diversity undertaken in *vegan* at each time point for test and control samples were significant at every time point (T_0_–T_3_, PERMANOVA, P < 0.001), indicative of standing compositional differences between test and control tanks prior to the addition of copepodids. However, R^2^ estimates did increase between test and control tanks over the course of infection, suggesting an increasingly important role of *L. salmonis* infection in explaining the variance between treatments as infection progressed (PERMANOVA, R^2^, T_0_:0.2608; T_1_:0.2726; T_2_:0.3351; T_3_:0.3492, p < 0.001 in all cases).

### Dominant microbial taxa, taxon associations and networks

**Figure 5:**
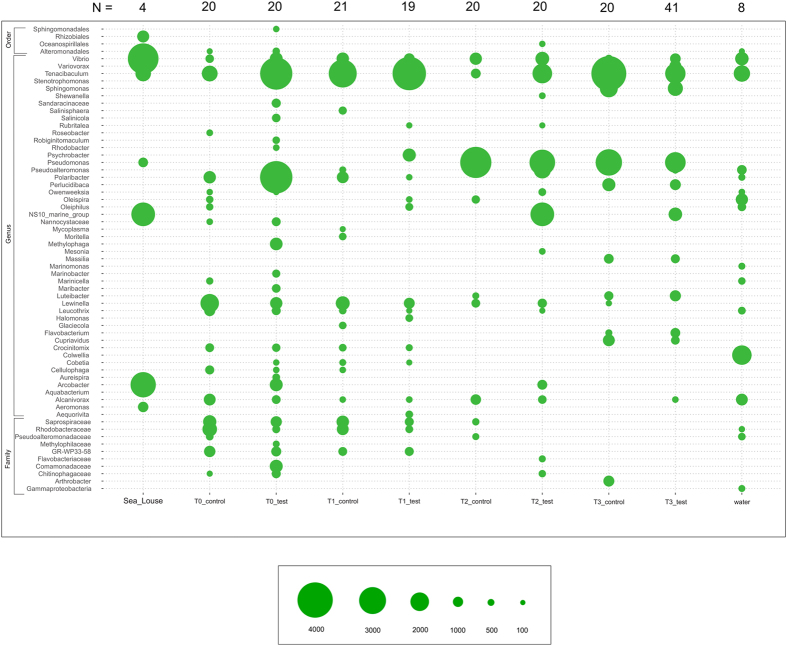
Taxonomic classifications and abundances of OTUs recovered among *Salmo salar, Lepeophtheirus salmonis* and water samples. The bubble shows mean abundance of core OTU taxomonic assignments (y axis, present in >85% of samples, represented by >100 sequences) in each sample group respectively (test (infected) vs control (uninfected) at each time point) on the x-axis. Variance associated with mean abundances are included in Supplementary Data.

**Figure 6:**
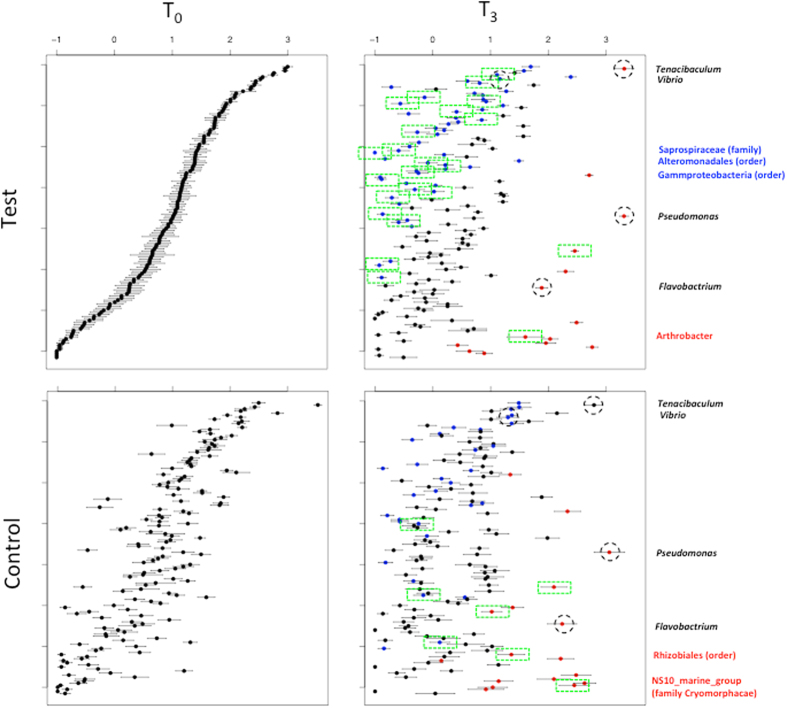
Comparisons of mean abundance of bacterial taxa between infected and uninfected fish. Plots show log abundance of different taxa (y axis) compared between T_0_ and T_3_ of all control (A) and test (B) tanks, respectively (x axis). Error bars are +/− standard error. Based on a Kruskal-Wallis test, data point (closed circles) colours in T_3_ indicate where an OTU was significantly less abundant than at T_0_ (blue), more abundant (red) or not significantly different (black). Abundance differences between taxa in control (top) and test (bottom) treatments for T_0_ (left) - T_3_ (right) comparisons (that are still significant after Bonferroni correction) are marked up by green dashed boxes. Putative secondary pathogens are listed in black (and indicated by the black dashed circles). Listed in red are taxa that were more abundant at T_3_ of control or infected fish respectively. Listed in blue were taxa that are less abundant given the same criteria.

**Figure 7:**
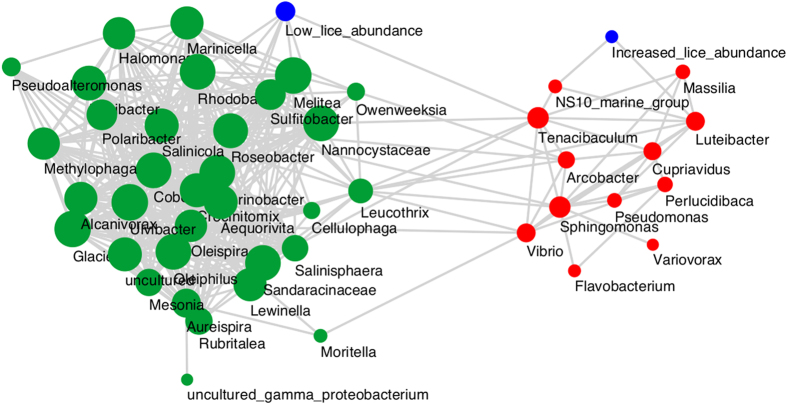
Network of bacterial taxa based on co-abundance of the 50 most abundant bacterial genera on all infected fish between samplings T_0_ and T_3_. The abundance of the sea lice on each fish has been used as a factor. Each node represents a taxon or louse abundance. An edge between two samples indicates a Spearman correlation index >0.7 between the two samples and a correlation p-value corrected with Bonferroni <0.05. The size of each node is proportional to the number of edges to which it is connected. The two main clusters are labeled green (putative commensal) and red (putative pathogens). High lice abundance correlations refer to taxa which are positively correlated with lice abundance (Spearman correlation >0.6), whereas low lice abundance correlations refer to taxa negatively correlated with lice abundance (Spearman correlation <−0.6).

At the genus level, *Tenacibaculum* was perhaps the most abundant taxon across all samples in the experiment, including mucus and water in both tests and controls (Fig. 5). *Tenacibaculum* was present but relatively less abundant in louse samples compared to other genera. Additional genera present at high abundances globally included *Vibrio, Pseudomonas* and *Lewinella. Vibrio* was particularly abundant among *L. salmonis* intestine samples, as was the genus *Arcobacter* and NS10_marine_group, a member of family Cryomorphaceae. To more robustly assess changes in taxon abundance in test and control tanks, we applied a Kruskal-Wallis test^15^. In view of standing variation present at T_0_ between infected and control fish, direct comparisons between treatments at T_3_ would be meaningless. Therefore, we compared taxon abundance in control and infected tanks respectively between T_0_ and T_3_ and noted differences between these two comparisons (Fig. 6). Genera significantly (P < 0.001 after Bonferroni correction) more abundant at T_3_ in infected fish but not controls included Rhizobiales and NS10_marine_group (family Cryomorphacae). Only *Arthrobacter* were more abundant at T_3_ in controls than in infected fish. Less abundant taxa in controls between T_0_ and T_3_ but not infected fish were individual OTUs within family Saprospiraceae, order Alteromonadales and order Gammaproteobacteria. The relative abundance of individual genera containing known salmonid pathogen species: *Tenacibaculum, Vibrio, Flavobacterium, Pseudomonas* was not higher among *L. salmonis* - infected fish at T_3_ as compared to the control T_0_–T_3_ comparison (Fig. 6). We also explored any correlation with individual OTUs and louse load in the larger cohort of infected fish. No significant negative associations were uncovered (bacterial taxa associated with low louse loads). However, two OTUs – one belonging to *Verrucomicrobia*, the other *Lewinella* were consistently associated with increasing louse load (P < 0.001 after Bonferroni correction) in all three tests applied. Consistent with Fig. 4, *Arcobacter*, presumably of louse origin, was also positively associated with louse load at T_3_. Network analysis, including louse load as a continuous variable, partitioned the 50 most abundant OTUs in infected fish into two correlated groups (Fig. 7), one large guild comprising mainly commensals, the other containing a number of putative pathogenic genera (*Pseudomonas, Tenanicibaculum, Flavobacterium*, among others). Importantly, significant associations were apparent between the commensal guild and lower louse load and the pathogenic guild and higher louse abundances on individual fish (Fig. 7). Thus, while individual associations between given microbial taxa and increasing louse abundance were limited – second order, multi-taxa associations were clearly at play.

## Discussion

Commensal microbiota may play a fundamental role in mediating host-parasite interactions (e.g. ref. 1, 2, 3). The aim of this study was to explore the impact of *L. salmonis* infection on the microbiota associated with Atlantic Salmon skin mucus in the context of salmon pathology, louse life-cycle stage (T_0–3_), and susceptibility to intense louse infections as well as secondary bacterial infections. We were successfully able to demonstrate the destabilizing influence that parasitism exerts on salmon skin microbiota. We did not demonstrate a link between louse infection and *individual* secondary pathogens. However, network analysis did reveal pathogenic and non-pathogenic guilds present within the communities of infect fish that correlated with high-intensity and low intensity infections respectively. We can thus conclude that perturbation of the mucosal microbiome may promote pathology via proliferation of endogenous pathogenic genera and/or via decreased colonization resistance to exogenous opportunists.

Numerous experimental studies have charted the detrimental impact of louse infection on marine phase Atlantic salmon in terms of basic morbidity and stress (e.g. ref. 16) as well as detailed immunological and transcriptional responses^17^,^18^. Our data generally corroborate these studies in terms of reduced fish performance in three out of four of our infected tanks. However, the limited time of exposure of the post-smolts to adult lice resulted in mass changes that were borderline with respect to controls. Mortality associated with louse load was not observed. Nonetheless, we did achieve our primary aim in obtaining intense *L. salmonis* loads in *S. salar* that developed through to adult stage (mean parasites per fish: 23.53), providing the opportunity to track microbial diversity over the time course of infection.

The composition of the *S. salar* associated intestinal microbiome is increasingly well understood in both wild^19^ and aquaculture^20^ settings. Furthermore, the relative contributions of environment and host to shaping euryhaline teleost gut microbial diversity have also been estimated^21^. Data concerning the epidmermal mucosal microbiome in salmonids are less common, especially in the marine phase. Boutin *et al*., 2013 have extensively characterized freshwater salmonid mucosal microbiota in brook char (*Salvelinus fontinalis*) in the context of emergent opportunistic infections and stress^22^,^23^. Dominant genera in our study (e.g. *Tenacibaculum, Lewinella, Vibrio*) were highly divergent with respect to those uncovered by Boutin *et al*., with the possible exception of *Pseudomonas* species^22^. Human skin microbiota are known to be among the most temporally unstable assemblages in the human body, as well as showing high levels of inter-individual variation^24^. The high degree of sharing apparent between environmental (principally water) and salmon skin microbiota stands in stark to sharing between environmental samples and *S. salar* gut microbiota^19^. It is also apparent that time (rather than infection status) is the major driver behind many differences one sees between microbial assemblages in this study (Figs 4 and 5). However, fluctuations in environmental microbiota did not seem to be the root cause of such differences. Instead, most water samples were associated with salmon mucus samples at T_0_–T_1_ only, while salmon mucus a samples T_2_&T_3_ were divergent and distinct from those in the water. It is not clear whether skin microbiota might eventually converge on a stable state with respect to time, or whether, like in other vertebrate systems, skin communities are continually subject to high levels of stochastic temporal change (e.g. ref. 24).

Sampling point (time) was not the only driver of microbiome community dynamics. Infection with *L. salmonis* did play an increasingly important role in defining microbial community identity as infection progressed, as revealed by multivariate analyses. In addition to community identity, we were able to demonstrate that community richness and beta-diversity were both impacted. ‘Destabilization’ of host-associated microbiota in comparison to healthy controls is a consistent feature of diseased states in both non-communicable (e.g. Crohns disease)^25^ and communicable disease (e.g. Giardia)^2^. The direction that these so-called ‘dysbioses’ take is a matter for debate. Simple reductions in microbial diversity and/or richness can be associated with conditions such as Crohns^26^. Directional shifts in community identity can also be detected in *Plasmodium*-infected mice^27^. Moreover, microbial co-occurrence networks shift in bowel cancer and changes in microbiome functional metabolic signatures can be detected in periodontitis^28^,^29^. The impact of such microbial dysbiosis on the host is less clear, and may indeed be either a primary, deterministic feature that allow opportunistic disease to occur or a secondary, neutral feature of primary pathogenesis with little more than diagnostic significance. Given the importance of secondary infections in the *L. salmonis* system, the destabilization of surface microbiota may, however, have a direct impact on host health – perhaps primarily via the declining ‘colonization resistance’ exerted by skin commensals that may result. Invasion ecologist Charles E. Elton first hypothesized that diverse communities might resist evasion more effectively that stable ones^30^. Various modifications of this argument linking aspects of microbial diversity to invasibility (i.e. colonization resistance) can be uncovered throughout the literature (reviewed in ref. 31). Fluctuating alpha and beta-diversity in infected fish did not significantly impact the abundance of putative pathogens in our study at individual level. For example OTUs of genus *Tenacibaculum* (to which *Tenacibaculum maritinum*, the etiological agent of salmon ulcerative tenacibaculosisis belongs)^32^ were abundant in almost all fish sampled, irrespective of whether or not they where infected with *L. salmonis*. Individual OTUs that were significantly associated with louse load among infected fish (one belonging to phylum Verrucomicrobia, the other classified as *Lewinella*) were not attributable to any known pathogen. OTUs found associated with sea lice intestines showed some interesting features. The capacity of *L. salmonis* to propagate disease agents has been the subject of some discussion in the literature (e.g. ref. 33). *Vibrio*, a genus comprising several major fish pathogens^6^, amongst other commensal taxa, was highly abundant in louse samples, although also present among fish and environmental samples in test and control tanks. One bacterial OTU (NS_10: Cryomorphaceae) was very clearly associated with louse infection and was amplified exclusively from lice intestines and test tanks T_2_ and T_3_. Whilst the importance of this the bacterium is not clear, the data suggests an ability to proliferate in the louse and transfer effectively from one host to another and a role as an indirectly transmitted pathogen cannot be ruled out.

Whilst associations between louse load and individual bacterial taxa do not suggest a clear link between parasite burden and the abundance specific secondary disease agents, network analyses were less equivocal. In line with previous work on microbial assemblages from salmonid skin mucus, co-occuring guilds of bacteria (respectively putative commensals or pathogens) persist whose relative abundance can be modulated by stress^22^ – in our case corresponding to parasite load. Establishing the role of such community dynamics in driving opportunistic disease or transmissible disease susceptibility is a crucial goal of future research. As such, maintaining stability in skin surface microbial assemblages via pre- pro- or syn-biotics may provide and effective means of mitigating disease in parasitized fish. Co-infection experiments are vital in this context, involving paired macro- and micro- pathogens to simulate the real world scenarios (e.g. ref. 11). Thus our study underlines the importance of taking a holistic approach that incorporates changing host, parasite and microbiome to appreciate their relative roles in modifying disease outcome.

## Materials and Methods

### Experimental procedures

Salmon post-smolt (mean mass at experiment outset 149 g +/− 13.1 g SE) from 50 salmon families were internally Passive Integrated Transponder (PIT) tagged and distributed randomly across six 1000 L tanks in a flow through system at the Fisheries and Oceans Canada marine facility St. Andrews Biological Station (St. Andrews, New Brunswick (NB), Canada). All fish handling and procedures were approved by DFO Maritimes & Gulf/CFIA Regional Animal Care Committee (File Number 14–13) and carried out under the direct supervision of a trained Department of Fisheries and Oceans Canada operative in strict compliance with regulations set out by the Canadian Council for Animal Care (http://www.ccac.ca/). Water conditions were maintained at 11–14 **°**C with a salinity of 30–33 g L^−1^. Each tank housed a maximum of 200 fish at a density under 40 kg m^−3^ and water quality parameters (temperature and oxygen) were monitored daily. Fish were fed with commercial salmon feed (2.5 mm) at 1–2% body weight per day and oxygen was added to maintain a saturation level between 90 and 105% (8–10 mg/l). Following an acclimation period of three weeks, four of the six tanks salmon were challenged with infective *L. salmonis* copepodids at a concentration of 40 copepodids per fish (8 copeodids L^−1^) for 1 hour. Copepodids were hatched from egg strings collected from gravid female lice gathered at a commercial salmon farm by technical staff from the Huntsman Marine Sciences Centre (HMSC), St. Andrews, NB, Canada. Water flow to the experimental exposure tanks was stopped just prior to addition of lice and fish were observed closely during the infection event. Jumping, flashing and behaviours such as rapid swimming were observed which is consistent with lice infection. After 1 hour, water flow was resumed and fish were not handled until the required sampling time point.

At 48 hours prior to infection (T_0_), 6 days (T_1_), 22 days (T_2_) and 35 (T_3_) after infection, bacterial community sampling was undertaken. Mucus samples comprised skin swabs along one full lateral surface of the fish (including the gill operculum). Samples from two control (Tank C1&C2 - uninfected) and two test (Tank Test_1&Test_2 - infected) tanks were taken at sample point T_0–3_. In addition, samples were taken from two further test tanks at T_3_ (Tank Test_3&Test_4, identical conditions to Test_1&Test_2) to provide further insight on the impact of adult lice. A single inflowing water bacterial community sample was taken per time point (10 litres filtered through a 0.2 μm filter). Biofilm samples were taken along the sides of each tank per time point also. During sampling, all fish in each tank were sedated using Aquacalm at 0.9 mg/l and 25 fish from each tank per time point were sampled randomly using individual sterilised soft-mesh nets to avoid cross-contamination and to avoid dislodging lice. Length (cm) and mass (g) were also recorded. Skin pH data for 10 fish were collected, while a blood sample for serum cortisol determination was collected for 5 fish per tank at time points T_1–3_. At day 35 (T_3_) all fish were euthanized with Tri-methanosuphonate (TMS) at 100–150 mg/l in individual nets to account for mobile lice loss and lice count data, weight length and sex were recorded. At T_3,_ 10 adult lice per tank were collected and treated with 0.1% hypochlorite solution for 30 minutes to remove adherent microbes, washed with microbe free water, pooled and frozen for gut microbial analysis.

### 16S rDNA amplicon sequence analysis

Mucus, environmental (biofilm, water) and louse samples were collected in sterile micro-centrifuge tubes and immediately stored in liquid nitrogen (−196 °C) until DNA extraction at the Institut de Biologie Intégrative et des Systèmes, at the Université Laval (Québec, QC). DNA was extracted from all samples using the Qiagen DNeasy blood and tissue kit according the manufacturers instructions. Amplification of the 16S rRNA V4 region was achieved with primers 519_f 5′-CAGCMGCCGCGGTAA-3′ and 785_r 5′-TACNVGGGTATCTAATCC-3′ using Takara *Taq* Polymerase (CloneTech, USA), and a final concentration of 1 pmol of each primer^19^. Reaction conditions were 95 °C for five minutes, followed by 30 °C cycles and of 95 °C for 30 seconds, 55 °C for 30 seconds and 72 °C for 30 seconds, followed by a final elongation step of 72 °C for 10 minutes. Each amplification was run in triplicate (technical replicates) and pooled to minimise PCR bias, purified using an AxyPrep™ Mag PCR Clean-Up Kit (Corning, USA). Sequence libraries were dual indexed using Illumina Nextera multiplex barcodes and sequenced in a single run on an Illumina MiSeq platform. V4 was chosen in the light of its widespread use to profile vertebrate-associated microbiota as well as its suitability for Illumina paired end sequence read lengths at the time of sequencing^34^.

Amplicon data were processed as described previously (ref. 19). Briefly, SICKLE^35^ was used for error screening (>Q30) and assembly of each paired end read into a single overlapping 290 bp fragment from the 16S rRNA V4 hypervariable region was achieved in PANDASeq^36^. Sequences were aligned against the *E. coli* 16S rRNA gene and trimmed in Mothur^37^ prior to operational taxonomic unit clustering in UPARSE at 97% identity^38^. Putatively chimeric OTUs were filtered out in reference to the genomes online database (GOLD v.5) in UCHIME^39^. Subsequently, the following steps were undertaken in QIIME^15^: after exclusion of chimeric OTUs, samples containing <13,700 reads were discarded and all samples were rarefied to an even depth of 13,700 reads. 13,700 represented the optimal minimum depth at which saturation was achieved while still including the maximum number of sample. OTUs with fewer than 100 reads or that only occurred in a single sample were filtered out as a step to improve accuracy and diversity estimates^40^.

### Statistical and diversity analyses

Fish mass and growth rate (where the same individuals were resampled at different time points – mean mass gain (g) day^−1^) were recorded throughout the experiment. Differences in mass between time points and between test (infected) and controls (uninfected) were plotted and assessed for significance using mixed models incorporating different tanks as a random effect in R using lme4 (*lmer*(Weight~Time_point*Test + (*1|Tank_Number*)) and tested for significance using a likelihood ratio test in the same package (*anova*(*null, model*))^41^. For the microbial samples themselves, Shannon diversity and Chao1 richness estimators were calculated for each rarefied sample in QIIME^15^. Mixed models were also applied to assess the distribution of variation in these parameters per treatment (fixed), tank (random) and sample point (fixed) using lme4 and lmertest^41^. To evaluate differences in community composition (beta-diversity), unweighted Unifrac distances were calculated and plotted^42^. Differences in beta-diversity between treatments and tanks were also assessed using mixed models in lme4^41^. Beta-diversity comparisons between all samples were also subjected to principal coordinates analysis, also in QIIME^15^. Differences in microbiome composition between test (Test tank 1 & Test Tank 2) and control (Control Tank 1 & Control 2) tanks at each time point (T_0–3_) were tested using a permutation-based multivariate analyses of variance (PERMANOVA) in ADONIS in the Vegan package in R^43^. OTU abundances, genus and order-level taxonomic classifications were calculated and plotted. Differential abundance of majority OTUs (i.e. comprising 95% of all samples) were compared between times T_0_ and T_3_ in control and infected fish treatments respectively and tested for significance using a non-parametric Kruskal-Wallis test in QIIME. Among infected fish from four tanks at T_3_, correlations were explored between microbial diversity and abundance and sea louse load as well as individual fish growth rate (mass (g) day^−1^) via several Bonferroni-corrected correlation tests in QIIME including: Pearson, Kendal and Spearman rank tests. Only consistently occurring OTUs across these measures were reported. Finally, network analysis was achieved in Cytoscape v.3.2.1 based on correlations between the relative abundance of the top 50 OTUs on the test fish (T_0_–T_3_) in relation to lice load. Spearman correlations and node weightings were calculated in the R packages *multtest, Hmisc*,*parallel* and *iterators*. Correlations were considered significant when the Spearman correlation value was >0.6 and the correlation p-value (corrected with Bonferroni) was <0.05.

## Additional Information

**How to cite this article**: Llewellyn, M. S. *et al*. Parasitism perturbs the mucosal microbiome of Atlantic Salmon. *Sci. Rep.* **7**, 43465; doi: 10.1038/srep43465 (2017).

**Publisher's note:** Springer Nature remains neutral with regard to jurisdictional claims in published maps and institutional affiliations.

## Supplementary Material

Supplementary Information
