# Supplementary material for: Parasitism perturbs the mucosal microbiome of Atlantic Salmon
Source: Sci Rep. 2017 Mar 7;7:43465. doi: 10.1038/srep43465 (PMC5339869; doi:10.1038/srep43465)
Supplement: Supplementary Information [file srep43465-s1.pdf]

Supplementary information for:

Parasitism perturbs the mucosal microbiome of Atlantic Salmon

Authors: **Llewellyn, M.S.*[1], Leadbeater, S.*[2] Garcia, C.[3], Sylvain F-E[3], Custodio, M.[4] Ang, K. P. [5] Powell, F.,[5] Carvalho, G. R.,[6] Creer, S.[6] Elliot, J.[5] Derome, N.[3]**

Figure S1 – Histogram of louse densities per infected fish across all tanks

Figure S2 – Box plot of louse infection intensities in each test tank

Figure S3 – Chao rarefaction curves demonstrating saturation across all samples in the dataset. Rarefactions were undertaken in ten steps between 10 - 13,700 reads for each sample.

Table S1 – Variances associated with bubble plot in Figure 5.

Table S2 - Summary table of samples subjected to 16S rRNA amplicon metabarcoding in this study

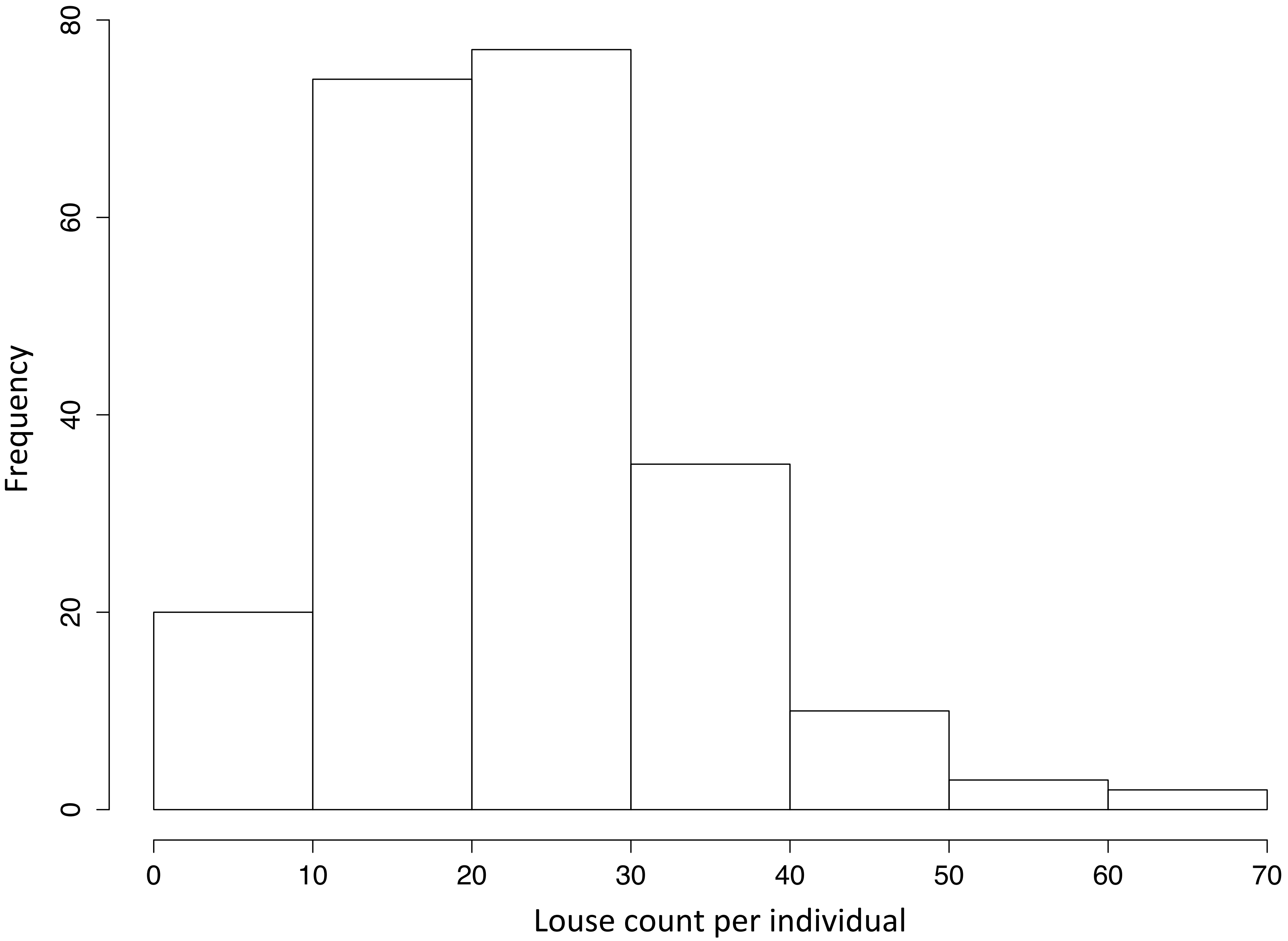

Frequency
0
20
40
60
80
0
10
20
30
40
50
60
70
Louse count per individual

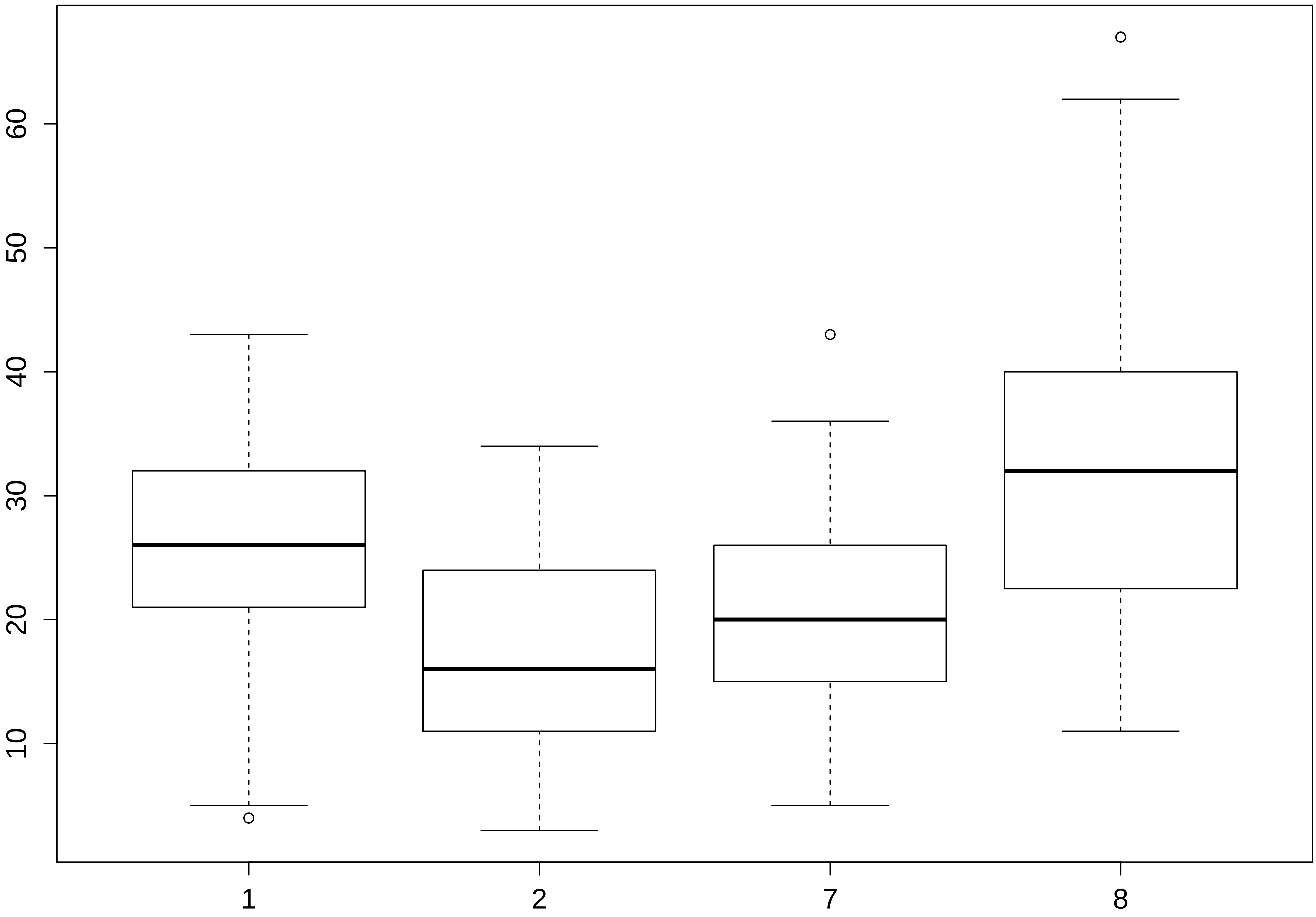
10
20
30
40
50
60
1
2
7
8

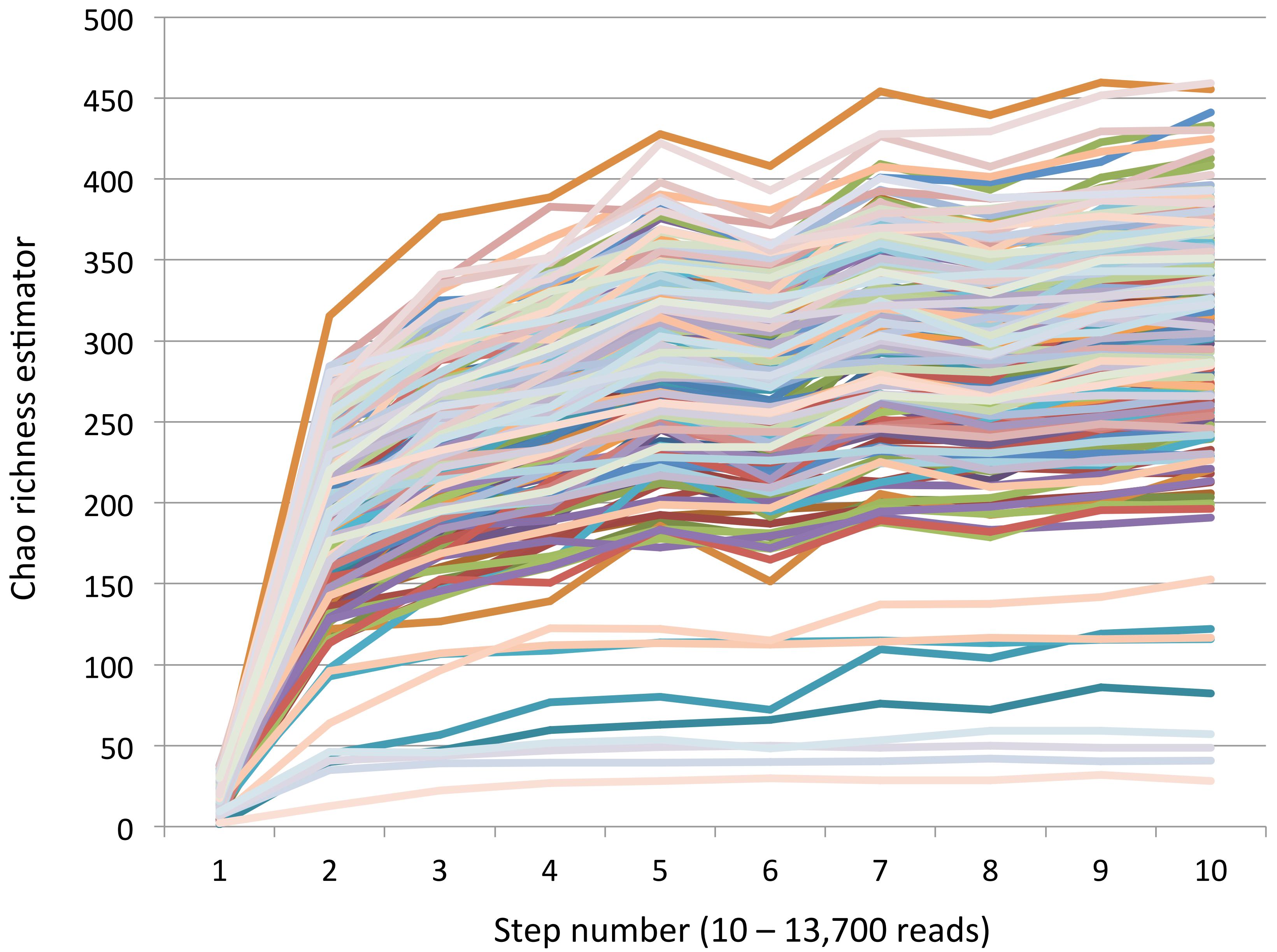
Chao richness estimator
500
450
400
350
300
250
200
150
100
50
0
1
2
3
4
5
6
7
8
9
10
Step number (10 – 13,700 reads)

class	Sample type	Variance
g__Aeromonas	Sea_louse	34.51599869
g__Aquabacterium	Sea_louse	368.9582901
g__Arcobacter	Sea_louse	20815.61131
g__NS10_marine_group	Sea_louse	2533.227466
g__Pseudomonas	Sea_louse	99.40135955
g__Tenacibaculum	Sea_louse	98.40476122
g__Vibrio	Sea_louse	40907.75889
o__Rhizobiales	Sea_louse	14.04987569
g__Alcanivorax	T3_test	545.2926659
g__Cupriavidus	T3_test	219.8622354
g__Flavobacterium	T3_test	270.0045411
g__Luteibacter	T3_test	2.606247577
g__Massilia	T3_test	182.1680063
g__NS10_marine_group	T3_test	211.4982472
g__Perlucidibaca	T3_test	8.912437412
g__Pseudoalteromonas	T3_test	412.2079135
g__Pseudomonas	T3_test	968.1851343
g__Sphingomonas	T3_test	744.5893041
g__Stenotrophomonas	T3_test	30.89067074
g__Tenacibaculum	T3_test	6351.373468
g__Variovorax	T3_test	8.601000433
g__Vibrio	T3_test	112.310552
f__Arthrobacter	T3_control	7.043207026
g__Cupriavidus	T3_control	7.204142734
g__Flavobacterium	T3_control	464.5628838
g__Lewinella	T3_control	589.9735277
g__Luteibacter	T3_control	157.6822663
g__Massilia	T3_control	93.23944624
g__Perlucidibaca	T3_control	124.8790592
g__Pseudomonas	T3_control	4653.37866
g__Sphingomonas	T3_control	2849.118323
g__Stenotrophomonas	T3_control	174.55833
g__Tenacibaculum	T3_control	97057.24819
g__Variovorax	T3_control	472.5547288
g__Vibrio	T3_control	330.9381694
f__Chitinophagaceae	T2_test	418.5815531
f__Flavobacteriaceae	T2_test	532.5915405
g__Alcanivorax	T2_test	238.8202084
g__Arcobacter	T2_test	80.08855592
g__Leucothrix	T2_test	616.8483395
g__Lewinella	T2_test	127.7663204
g__Mesonia	T2_test	527.1939327
g__NS10_marine_group	T2_test	2811.722732
g__Owenweeksia	T2_test	357.0402792
g__Pseudoalteromonas	T2_test	2112.079618

g__Pseudomonas	T2_test	4521.148359
g__Rubritalea	T2_test	620.7363788
g__Shewanella	T2_test	520.0398054
g__Tenacibaculum	T2_test	608.5749013
g__Vibrio	T2_test	37.22924062
o__Oceanospirillales	T2_test	574.8847852
f__Pseudoalteromonadacea	T2_control	474.7270017
f__Saprospiraceae	T2_control	542.8618863
g__Alcanivorax	T2_control	28.29972356
g__Lewinella	T2_control	217.5473668
g__Luteibacter	T2_control	485.0008757
g__Oleispira	T2_control	273.8747098
g__Pseudoalteromonas	T2_control	-0.577991038
g__Pseudomonas	T2_control	5425.246339
g__Tenacibaculum	T2_control	85.81144484
g__Vibrio	T2_control	115.0982056
f__GR-WP33-58	T1_test	177.9650984
f__Rhodobacteraceae	T1_test	477.0005997
f__Saprospiraceae	T1_test	180.0604548
g__Aequorivita	T1_test	374.9904662
g__Alcanivorax	T1_test	534.3968406
g__Cobetia	T1_test	616.8483395
g__Crocinitomix	T1_test	474.7270017
g__Halomonas	T1_test	368.9582901
g__Leucothrix	T1_test	609.1088462
g__Lewinella	T1_test	151.8352216
g__Oleiphilus	T1_test	339.5291165
g__Oleispira	T1_test	534.3968406
g__Polaribacter	T1_test	567.4135846
g__Psychrobacter	T1_test	46.56271423
g__Rubritalea	T1_test	593.7762012
g__Tenacibaculum	T1_test	79713.31391
g__Vibrio	T1_test	56.75018992
f__GR-WP33-58	T1_control	201.6847502
f__Rhodobacteraceae	T1_control	104.1508433
f__Saprospiraceae	T1_control	48.04049866
g__Alcanivorax	T1_control	556.2982472
g__Cellulophaga	T1_control	545.2926659
g__Cobetia	T1_control	495.3845058
g__Crocinitomix	T1_control	371.9682806
g__Glaciecola	T1_control	384.1301939
g__Leucothrix	T1_control	405.8830544
g__Lewinella	T1_control	11.2485533
g__Moritella	T1_control	390.2843213
g__Mycoplasma	T1_control	616.8483395
g__Polaribacter	T1_control	58.34345642

g__Pseudoalteromonas	T1_control	491.9111007
g__Salinisphaera	T1_control	300.3770541
g__Tenacibaculum	T1_control	7643.420991
g__Vibrio	T1_control	53.24460859
f__Chitinophagaceae	T0_test	512.9344585
f__Comamonadaceae	T0_test	107.9667693
f__GR-WP33-58	T0_test	66.63499215
f__Methylophilaceae	T0_test	488.4498907
f__Rhodobacteraceae	T0_test	420.7169397
f__Saprospiraceae	T0_test	157.2890487
g__Alcanivorax	T0_test	208.4098438
g__Arcobacter	T0_test	106.3426135
g__Aureispira	T0_test	357.0402792
g__Cellulophaga	T0_test	556.2982472
g__Cobetia	T0_test	578.6386781
g__Crocinitomix	T0_test	266.1618114
g__Leucothrix	T0_test	180.0604548
g__Lewinella	T0_test	29.72558853
g__Maribacter	T0_test	226.8800119
g__Marinobacter	T0_test	305.8238644
g__Methylophaga	T0_test	67.10016249
g__Nannocystaceae	T0_test	190.7201635
g__Owenweeksia	T0_test	605.2573923
g__Polaribacter	T0_test	5895.171922
g__Rhodobacter	T0_test	538.0165872
g__Robiginitomaculum	T0_test	399.6069758
g__Salinicola	T0_test	238.8202084
g__Sandaracinaceae	T0_test	159.6556715
g__Tenacibaculum	T0_test	14397.70616
g__Vibrio	T0_test	26.11174411
o__Alteromonadales	T0_test	399.6069758
o__Sphingomonadales	T0_test	567.4135846
f__Chitinophagaceae	T0_control	616.8483395
f__GR-WP33-58	T0_control	-0.75650867
f__Pseudoalteromonadacea	T0_control	368.9582901
f__Rhodobacteraceae	T0_control	-0.095296764
f__Saprospiraceae	T0_control	143.5832572
g__Alcanivorax	T0_control	5.119949009
g__Cellulophaga	T0_control	171.7522
g__Crocinitomix	T0_control	208.4098438
g__Leucothrix	T0_control	5.769333018
g__Lewinella	T0_control	300.0018153
g__Marinicella	T0_control	516.4810343
g__Nannocystaceae	T0_control	530.7892891
g__Oleiphilus	T0_control	418.5815531
g__Oleispira	T0_control	396.4872292

g__Owenweeksia	T0_control	563.696277
g__Polaribacter	T0_control	85.32708486
g__Roseobacter	T0_control	512.9344585
g__Tenacibaculum	T0_control	1072.975333
g__Vibrio	T0_control	311.3194552
o__Alteromonadales	T0_control	624.6366132
c__Gammaproteobacteria	Water	488.4498907
f__Pseudoalteromonadacea	Water	387.20116
f__Rhodobacteraceae	Water	586.1830494
f__Rhodobacteraceae	Water	582.4047662
g__Alcanivorax	Water	4.739695629
g__Colwellia	Water	4814.570266
g__Leucothrix	Water	357.0402792
g__Marinicella	Water	409.0393864
g__Marinomonas	Water	488.4498907
g__Oleiphilus	Water	238.8202084
g__Oleispira	Water	26.11174411
g__Owenweeksia	Water	534.3968406
g__Polaribacter	Water	471.3267673
g__Pseudoalteromonas	Water	100.9723257
g__Tenacibaculum	Water	145.8358145
g__Tenacibaculum	Water	197.2623302
g__Vibrio	Water	36.72088172
g__Vibrio	Water	187.1935088
g__Vibrio	Water	624.6366132
o__Alteromonadales	Water	628.5490427

		Tank ID	Time point T_0	T_1	T_2	T_3
Salmon mucus	Control	Control Tank 1	10	11	10	12
		Control Tank 2	10	10	10	8
	Total control		20	21	20	20
	Test	Test Tank 1	10	9	10	11
		Test Tank 2	10	10	10	10
		Test Tank 4	0	0	0	10
		Test Tank 3	0	0	0	10
	Total test		20	19	20	41
L. salmonis	Test	Test Tank 1	0	0	0	1 (pooled)
		Test Tank 2	0	0	0	1 (pooled)
		Test Tank 4	0	0	0	1 (pooled)
		Test Tank 3	0	0	0	1 (pooled)
	Total lice					4
Water Biofilm		Shared Water	2	2	2	2
		Control Tank 1	1	1	1	1
		Control Tank 2	1	1	1	1
		Test Tank 1	0	1	1	0
		Test Tank 2	1	1	1	0
		Test Tank 4	0	0	0	0
		Test Tank 3	0	0	0	1
	Grand total					126
